# Longitudinal changes in resting-state EEG eyes-closed/open delta differentiation during an Antarctic expedition

**DOI:** 10.3389/fnins.2026.1944325

**Published:** 2026-10-02

**Authors:** Weiting Yang, Haoxin Tian, Guangming Xie, Ying Xu, Qinwen Wang, You Yin, Wuchao Liu, Yanyan Jiang

**Affiliations:** 1 Department of Neurology, Shanghai East Hospital, School of Medicine, Tongji University, Shanghai, China; 2 Department of Neurology, Shanghai General Hospital, Shanghai Jiao Tong University School of Medicine, Shanghai, China; 3 Research Center for Translational Medicine, Shanghai East Hospital, School of Medicine, Tongji University, Shanghai, China; 4 Institute of Basic Medicine, North Sichuan Medical College, Nanchong, Sichuan, China; 5 Department of Neurology, Second Affiliated Hospital (Shanghai Changzheng Hospital) of Naval Medical University, Shanghai, China; 6 Department of Neurology and Neurological Rehabilitation, Shanghai Yangzhi Rehabilitation Hospital (Shanghai Sunshine Rehabilitation Center), Tongji University School of Medicine, Shanghai, China

**Keywords:** Antarctic polar night, eyes-closed/open delta differentiation, linear mixed-effects model, resting-state EEG, state reactivity

## Abstract

Antarctic polar night brings extreme light deprivation, prolonged isolation and severe circadian disruption, yet longitudinal resting-state electroencephalography (EEG) signatures reflecting neurophysiological alterations in this harsh environment remain poorly characterized. In this exploratory longitudinal research, resting-state EEG recordings with eyes open (EO) and eyes closed (EC) were acquired from 16 Antarctic expeditioners at four mission stages: pre-departure, pre-polar-night, early post-polar-night, and austral summer. Linear mixed-effects models with false discovery rate correction were applied to analyze delta eyes-closed/open differentiation (the EC–EO delta log-power difference), delta sub-bands, aperiodic spectral features, entropy, EEG microstates and delta-band functional connectivity. The delta EC–EO difference exhibited consistent, progressively negative shifts in parietal, frontal, whole-brain and occipital regions across the expedition timeline, with the most pronounced shifts observed in recordings obtained during and after the polar-night period; this effect spanned all slow, mid and fast delta sub-bands. Secondary mild temporal variations were observed in aperiodic spectral metrics, occipital sample entropy and global delta connectivity, whereas EEG microstates displayed no significant longitudinal alterations. Collectively, the delta EC–EO difference emerges as a candidate EEG index of resting-state condition differentiation in the low-frequency range, pending validation in larger studies with synchronized behavioral, physiological, and environmental measures.

## Introduction

Antarctic expeditions offer a unique natural model for investigating human neurophysiology under extreme environmental and operational conditions (Roalf et al., 2025). Expedition members experience prolonged isolation, restricted social and physical environments, high task demands, low temperature exposure, and marked changes in natural light availability (Tassino et al., 2016; Tortello et al., 2023). The polar-night period is particularly relevant for neurophysiological research because reduced or absent daylight may be associated with changes in circadian regulation (Kawasaki et al., 2018), sleep-wake organization, fatigue, and resting-state brain activity (Pattyn et al., 2018; Silva et al., 2019). At the same time, such field studies are constrained by small sample sizes, difficult acquisition conditions, and limited opportunities for repeated measurement. These constraints make a cautious exploratory longitudinal framework more appropriate than a confirmatory mechanistic design (Mairesse et al., 2019).

Resting-state EEG is a non-invasive signal modality with high temporal resolution and practical feasibility in field settings (Strijbis et al., 2022). Moreover, resting-state EEG has been increasingly used for cognitive state assessment and characterization, with adaptive spatiotemporal encoding models accurately predicting cognitive levels from resting-state recordings (Sun et al., 2024). Compared with cross-sectional measurements, longitudinal resting-state EEG can characterize within-participant changes across an expedition timeline and reduce the influence of between-participant baseline variability (Liu et al., 2024). The contrast between EO and EC resting-state conditions provides a useful index of state reactivity or resting-state condition differentiation. Although alpha reactivity is frequently emphasized in resting-state EEG, low-frequency activity may also vary with vigilance, arousal regulation, and broad spectral background structure (Celli et al., 2022; Garakh et al., 2024). Therefore, EO/EC difference measures may provide more interpretable information about state-dependent modulation than absolute power in a single resting condition. Low-frequency (delta) power is closely coupled to arousal level and sleep pressure (Lendner et al., 2020), and the eyes-closed-to-eyes-open transition engages widespread cortical-state changes that extend beyond the alpha rhythm (Barry et al., 2007). Because delta-band eyes-closed/open differentiation may index state-dependent low-frequency modulation, it was selected as the primary endpoint of the present study.

Existing studies of Antarctic expeditions have documented disruptions in sleep architecture, circadian rhythmicity, and cognitive performance across the polar-night period, but investigations of resting-state EEG signatures remain limited and inconsistent. Most prior EEG work in extreme environments has focused on absolute spectral power in a single resting state or on sleep EEG patterns, with relatively few studies examining state reactivity indexed by the contrast between eyes-open and eyes-closed conditions. Specifically, although low-frequency delta activity is known to vary with arousal level and sleep pressure, the longitudinal trajectory of delta-band eyes-closed/open differentiation during prolonged light deprivation and isolated confinement remains largely uncharacterized. Furthermore, it remains unclear whether such state-differentiation metrics would be more sensitive to extreme-environment effects than conventional absolute power measures, and whether they reflect frequency-specific changes or broader shifts in the aperiodic spectral background. This gap limits the ability to develop sensitive neurophysiological indices for monitoring brain state regulation in isolated, light-deprived populations. Establishing whether EEG state-differentiation metrics change longitudinally in this setting, and whether such changes are frequency-specific or reflect broader shifts in spectral background, is therefore a prerequisite for evaluating their potential as monitoring indices.

EEG changes observed during an Antarctic expedition should not be attributed to a single mechanism without supporting covariates (Moraes et al., 2023). Light exposure, sleep, fatigue, mood, operational workload, seasonal timing, and isolation may all contribute to resting-state EEG variation (Chmiel and Kurpas, 2026; Folgueira et al., 2019). In a small longitudinal expedition cohort, statistical effects can identify time-related patterns, but they cannot establish causal effects of specific environmental factors. For this reason, the present manuscript frames the work as an exploratory longitudinal resting-state EEG study and focuses on conservative description of EEG changes across the expedition timeline. Accordingly, the terms adaptation and biomarker are deliberately avoided when describing the present results; the findings are instead framed as longitudinal changes in resting-state EEG reactivity whose physiological interpretation requires further validation.

To address the identified research gap, this study tested three a priori hypotheses. First, as the primary hypothesis, delta-band eyes-closed/open differentiation (the delta EC–EO difference) would show longitudinal changes across the expedition, with the most pronounced alterations expected between the pre-polar-night assessment and the assessments obtained during and after the polar-night period. Second, these changes would span multiple delta sub-bands and would be statistically associated with aperiodic spectral features, reflecting both frequency-specific and broad-spectrum shifts. Third, other EEG metrics including complexity/entropy measures, microstate parameters, and functional connectivity would show weaker or less consistent longitudinal effects relative to the delta EC–EO difference.

The primary analysis focused on the delta EC–EO difference. This metric was defined as eyes-closed minus eyes-open delta log power, that is, EC delta log power minus EO delta log power. This endpoint is not equivalent to EC delta power alone and should not be interpreted as a direct increase in delta activity during the polar night (He et al., 2023). A more appropriate interpretation is that delta-band eyes-closed/open differentiation changed across the expedition timeline, with more evident differences during and after the polar-night period.

In addition to the primary analysis of the delta EC–EO difference, the study examined delta sub-bands, aperiodic spectral parameters, complexity/entropy measures, EEG microstates, and delta-band functional connectivity as secondary or exploratory feature families (Bandt and Pompe, 2002; Donoghue et al., 2020; Richman and Moorman, 2000). This hierarchical approach preserves a focused primary narrative while reporting a broader EEG feature space transparently. The goal was to provide an initial, methodologically explicit framework for future extreme-environment EEG monitoring studies that can incorporate larger samples, higher-density EEG, and behavioral or physiological covariates.

## Methods

### Participants and study design

This exploratory longitudinal study analyzed resting-state EEG data from 16 healthy adult male, right-handed members (mean age 37.8 ± 7.7 years, range 28–52 years) of China's 39th Antarctic Research Expedition who wintered at Zhongshan Station (69°22′S, 76°22′E). All participants had no history of neuropsychiatric disorders, chronic diseases, or long-term use of sleep-related medications, and had completed systematic pre-departure training in ice and snow environments. The expedition departed from Shanghai on October 31, 2022, arrived in Antarctica on November 22, 2022, and returned to Shanghai on January 16, 2024. EEG was collected at four repeated timepoints aligned with the expedition timeline: T1 (pre-departure baseline) was collected 1-2 weeks before departure, in mainland China; T2 (pre-polar-night) was collected approximately 1 month before the onset of polar night; T3 (early post-polar-night) was collected within 1 month after the end of polar night; and T4 (austral summer) was collected approximately 3 months after the end of polar night, before the return voyage. The polar-night period lasted from May 28 to July 17, 2023 (51 days), and all participants experienced the full polar-night cycle. The four measurement phases thus corresponded to the pre-departure phase in Shanghai (T1, October–November 2022), the pre-polar-night phase at Zhongshan Station (T2, April–May 2023), the early post-polar-night phase (T3, August 2023), and the austral-summer phase (T4, October–November 2023).

The intervals between consecutive timepoints were not fixed but followed the expedition timeline: from the recording dates, the mean interval was approximately 178 days between T1 and T2, 102 days between T2 and T3, and 83 days between T3 and T4 (ranges: 152–201, 83–109, and 75–94 days, respectively). All recordings were performed between 9:00 A.M. and 11:00 A.M. local time to minimize diurnal variation in EEG features. At each timepoint, each participant was scheduled to complete both EO and EC resting-state recordings. The theoretical data structure was therefore 16 participants × 4 timepoints × 2 conditions = 128 EEG files. At each session, participants were recorded in a supine position in a quiet, dimly lit room. Each recording began with an eyes-open (EO) resting-state block of at least 20 min, during which participants were instructed to remain awake; if any signs of drowsiness were observed, staff stimulated the participant to maintain wakefulness. Upon completion of the EO block, participants were instructed to close their eyes and remain in a resting state, and eyes-closed (EC) EEG was recorded for an additional 10 min. EEG signals were recorded with a portable 32-channel system equipped with silver/silver chloride (Ag/AgCl) electrodes (Arc Essentia, USA) at a sampling rate of 250 Hz. The EC block immediately followed the EO block within the same recording session, without a systematic rest interval between the two conditions.

### EEG files, channels, and regional definitions

EEG data were stored in EEGLAB ‘.set’ format and read using MNE-Python. File names followed the structure ‘S{subject}_{timepoint}_EEG_open_eyes.set’ or ‘S{subject}_{timepoint}_EEG_closed_eyes.set’. Of the recorded channels, analyses used 16 scalp electrodes from the 10–20 system: FP1, FP2, F7, F3, F4, F8, T3, C3, C4, T4, T5, P3, P4, T6, O1, and O2. For compatibility with the MNE standard montage, FP1/FP2 were mapped to Fp1/Fp2, and T3/T4/T5/T6 were mapped to T7/T8/P7/P8 for topographic visualization using the ‘standard_1020’ montage.

Regional summaries were computed using predefined electrode groups. The frontal region included FP1, FP2, F7, F3, F4, and F8; the central region included C3 and C4; the parietal region included P3 and P4; the temporal region included T3, T4, T5, and T6; the occipital region included O1 and O2; and the whole-brain region included all 16 electrodes. Regional grouping was based on standard functional-anatomical divisions of the 10–20 system, consistent with prior Antarctic EEG studies to ensure comparability across reports.

### Quality control and preprocessing

All EEG signals were re-referenced to the average of bilateral mastoid electrodes (M1/M2) before preprocessing. The signals were bandpass filtered from 0.5 Hz to 45 Hz using a zero-phase finite impulse response (FIR) filter, and a 50 Hz notch filter was applied to remove line noise. Ocular artifacts were identified and removed using independent component analysis (ICA) as implemented in MNE-Python. Myogenic and movement artifacts were detected using an amplitude threshold of ±100 μV; epochs exceeding this threshold in any channel were excluded from further analysis. Remaining bad channels were interpolated using spherical spline interpolation. A final manual visual inspection was performed by an experienced EEG technician to confirm artifact removal quality.

Quality control first assessed file availability, readability, channel completeness, epoch count, sampling rate, single-epoch duration, total recording duration, and a coarse artifact ratio. The coarse artifact ratio was based on an absolute amplitude threshold of 120 microvolts, used only for initial rapid screening. An epoch was marked as potentially abnormal if any channel at any timepoint exceeded this threshold. A coarse spectral slope of the channel-averaged signal power between 1 and 40 Hz in log-log coordinates was also computed as a signal-quality and spectral-shape check.

Among the 128 expected files, 128 were found. Of these, 127 files were readable and one file could not be read. All 127 readable files matched the expected 16-channel layout. Based on quality-control labels, 116 files were marked as usable and 11 files were marked as having a low epoch count. The median total duration among readable analysis files was 188.0 s, and the mean coarse abnormal-epoch ratio was 0.0. The 11 low-epoch-count recordings comprised nine eyes-open files (S1_T2, S1_T4, S4_T1, S5_T3, S10_T1, S12_T1, S12_T3, S14_T1, and S16_T4; 3–18 usable epochs) and two eyes-closed files (S1_T1 and S16_T1; 4–8 usable epochs); the single unreadable file was S12_T4_EO. A prespecified minimum-epoch criterion of 20 usable epochs per condition (i.e., ≥80 s of artifact-free data) was adopted as a data-quality threshold, based on previous evidence that reliable spectral estimates require a sufficient amount of artifact-free data (Gudmundsson et al., 2007). Because the primary endpoint of this study is a within-participant EC–EO contrast, both conditions at a given participant-timepoint are required to compute the difference. To avoid discarding an entire observation when only one condition fell below the quality threshold, all 63 readable participant-timepoint pairs were retained for the primary difference analysis, including the 11 pairs in which one condition had been flagged as having a low epoch count. This decision preserves the paired longitudinal structure, and maximum-likelihood mixed-effects modeling accommodates the resulting heterogeneity in estimate precision; the influence of the shortest recordings on the reported results is considered in the Limitations.

The primary analysis of the delta EC–EO difference included 63 participant-timepoint observations (from 16 participants across 4 timepoints, with one EO file unreadable). For functional connectivity analyses, the number of included observations ranged from 61 to 63 depending on the metric (EC PLV region-pair models: 61; global and selected dwPLI models: 63), reflecting metric-specific data requirements (e.g., files with insufficient epochs for stable phase estimation or incomplete region-pair coverage). Linear mixed-effects models were estimated using maximum likelihood, which accommodates unbalanced data and missing observations without imputation, using all available data points. The data acquisition protocol and quality-control outcomes are summarized in Table 1.

**Table 1:** Study design, EEG data structure, timepoints, and resting-state conditions.

Item	Value
Study design	Longitudinal repeated-measures resting-state EEG study
Participants	16 Antarctic expedition members
Timepoints	T1 baseline (pre-departure); T2 pre-polar-night; T3 early post-polar-night; T4 austral summer
Conditions	Eyes open (EO) and eyes closed (EC)
Expected data matrix	16 participants x 4 timepoints x 2 conditions = 128 EEG files
Found files	128/128
Readable files in QC manifest	127/128
Sampling rate	250 Hz
Epoch length	4.0 s; 1000 samples
Channels	16 scalp EEG channels
Amplitude rejection rule	Epoch rejected if any channel exceeded +/−100 uV after preprocessing
Condition-level usable data	eyes closed (all readable); datasets = 64; retained epochs = 3216; median epochs/file = 47.5; range = 4–116
Condition-level usable data	eyes open (all readable); datasets = 63; retained epochs = 2850; median epochs/file = 47; range=3–135
Minimum usable epochs	20 epochs (≥80 s artifact-free data per condition)
Recordings flagged below threshold	11 (EO: 9 files, 3–18 epochs; EC: 2 files, 4–8 epochs)
Primary EC–EO difference analysis	63 participant-timepoint pairs (both conditions readable)

### Spectral power and the delta EC–EO difference

Spectral power was estimated using Welch's method. Power spectral density (PSD) was computed for each epoch and channel over 0.5–45 Hz. The ‘n_fft’ parameter was set to ‘min (1024, n_times)’, ‘n_per_seg’ was set to ‘min (512, n_times)’, and ‘n_overlap’ was set to ‘min (256, max (0, n_times/4))’. Arithmetic averaging was used. PSD values were first averaged across epochs and then summarized at the channel, region, and frequency-band levels.

Canonical frequency bands were defined as delta (0.5–4 Hz), theta (4–8 Hz), alpha (8–13 Hz), beta (13–30 Hz), and gamma (30–45 Hz). Band power was computed by integrating PSD over the target frequency range and dividing by the bandwidth. To reduce scale skewness and improve model residual behavior, the primary band-power measures were expressed as natural log power, ‘log (power + 1e-20)’.

The primary spectral endpoint was the delta EC–EO difference, defined as EC delta log power minus EO delta log power within the same participant, timepoint, and brain region. To decompose this difference, EC and EO delta log power were additionally modeled separately as functions of timepoint using the same linear mixed-effects model framework as the primary analysis. Other frequency-band EO/EC differences were also computed to visualize the spatial distribution of condition-related spectral differences across timepoints.

### Delta sub-bands and aperiodic spectral features

To further characterize the internal structure of the delta range, delta was divided into total delta (0.5–4 Hz), slow delta (0.5–1 Hz), mid delta (1-2 Hz), and fast delta (2–4 Hz). The delta band was subdivided following standard conventions in arousal and sleep neurophysiology, to test whether the observed effects were confined to a specific narrow frequency range or distributed across the full delta spectrum. Each sub-band was computed using the same PSD integration procedure as the canonical frequency bands, followed by log transformation and EO/EC difference calculation.

Aperiodic spectral parameters were estimated using FOOOF. The primary model was fit over 1–40 Hz using fixed aperiodic mode, a peak-width limit of 0.5–12.0 Hz, a maximum of 6 peaks, a minimum peak height of 0.0, and a peak threshold of 2.0. These parameters were selected based on standard settings for resting-state EEG across the adult lifespan, with peak width limits optimized to separate oscillatory peaks from the 1/f aperiodic background in the 1–40 Hz range. For each EO and EC file, the aperiodic offset, aperiodic exponent, model R2, and fitting error were extracted. Alpha peak parameters were extracted within 8–13 Hz, retaining the center frequency, peak power, and bandwidth of the highest alpha peak.

### Complexity, entropy, EEG microstates, and functional connectivity

Complexity and entropy metrics were computed separately for frontal, central, parietal, temporal, occipital, and whole-brain regions. For each region, included channels were first averaged, and all epochs were concatenated into a single regional time series. To reduce computational load and standardize feature estimation, the time series was downsampled to 50 Hz, truncated to at most 2,500 samples, demeaned, and standardized. Downsampling to 50 Hz is standard practice for low-frequency complexity metrics, as these measures are dominated by slow temporal dynamics; downsampling reduces computational redundancy without compromising estimation accuracy for delta-band and sub-delta features.

Permutation entropy used embedding dimension 3 and delay 1 and was normalized by ‘log2(3!)’. Sample entropy used embedding dimension 2, a tolerance of 0.2 times the sequence standard deviation, and Chebyshev-distance matching. Lempel-Ziv complexity was computed after binarizing the standardized sequence by its median and was normalized by ‘n/log2(n)’.

EEG microstate analysis was conducted to characterize the temporal dynamics of large-scale scalp potential topographies following standard analytical pipelines (Pascual-Marqui et al., 1995). Preprocessed, artifact-free EEG data were used for microstate extraction, with topographies sampled at local maxima of the global field power (GFP). Extracted topographies were clustered into four canonical microstate classes (A, B, C, and D) via a modified k-means algorithm, consistent with widely replicated resting-state EEG findings (Michel and Koenig, 2018). Group-level microstate templates were generated by averaging GFP-peak topographies across all participants and conditions and then back-fitted to individual EEG data; each time point was assigned to the template with the highest spatial correlation. Three core metrics, mean duration, occurrence, and coverage, were calculated for each microstate class (Britz et al., 2010). Metrics were computed separately for eyes-open (EO) and eyes-closed (EC) states, and EC–EO contrast values were derived. Importantly, microstate outcomes reflect only scalp-level potential dynamics and should not be interpreted as source-level network localization (Michel and Murray, 2012).

Functional connectivity analysis was restricted to the delta band, defined as 1.25–4.0 Hz. The lower bound of this connectivity delta band was set to 1.25 Hz rather than the 0.5 Hz used for spectral power, because with the 4-s epochs used for connectivity estimation, frequencies below 1.25 Hz contain fewer than five full oscillatory cycles per epoch, which is insufficient for stable phase-based connectivity estimates (Ermolova et al., 2021; Zimmermann et al., 2024). If an input file could be read directly as an epoched EEGLAB file, the original epochs were used. If a file could only be read as continuous raw data, it was segmented into non-overlapping 4.0-s epochs. Recordings shorter than 4.0 s were read as a single segment. Connectivity analyses required a minimum of two usable epochs per recording, applied as a practical lower bound for the phase-based multitaper estimator; in this dataset the median number of retained epochs per file was 47, so files entering the connectivity models typically contributed substantially more epochs than this lower bound. Recordings that failed file reading or epoch-segmentation requirements for a given metric were excluded from that metric's models, resulting in 61–63 included observations across connectivity models. Connectivity was estimated using multitaper frequency-domain connectivity with ‘mode=“multitaper”’, ‘faverage=True’, ‘mt_adaptive=False’, ‘mt_low_bias=True’, and ‘n_jobs=1’. The formal connectivity metrics were phase-locking value (PLV) and debiased weighted phase-lag index (dwPLI). Coherence, AEC, PLI, and conventional wPLI were not treated as formal statistical endpoints.

### Statistical analysis

All formal inferences used linear mixed-effects models. For each EEG outcome, timepoint was modeled as a categorical fixed effect and participant was modeled as a subject-specific random intercept. The model formula was ‘outcome ~ timepoint + (1 | subject)’. Timepoint was treated categorically, and no linear spacing from T1 to T4 was assumed. Models were estimated using maximum likelihood rather than REML to allow likelihood-ratio testing between the full model containing the timepoint fixed effect and an intercept-only null model.

Effect sizes were reported as follows. For the omnibus timepoint effect, a standardized effect size (Cramér's V) was computed from the likelihood-ratio χ^2^ statistic as V = √(χ / [N(k−1)]), where k = 4 timepoints and N is the number of observations; values of 0.1, 0.3, and 0.5 were interpreted as small, medium, and large, respectively (Cohen, 2013). For the planned *post hoc* contrasts, estimated differences, 95% confidence intervals, and the paired standardized effect size (Cohen's dz) are reported, with Benjamini–Hochberg FDR correction applied across the four contrasts within each region. The main effect of timepoint was tested using a likelihood-ratio test. The test statistic was computed as twice the log-likelihood difference between the full and null models and evaluated using a chi-square distribution with degrees of freedom corresponding to the model difference. Model optimization prioritized ‘lbfgs’; if convergence failed, ‘powell’ was attempted with a maximum of 500 iterations. Multiple comparisons were controlled using Benjamini-Hochberg FDR correction within each feature family. For the primary delta EC–EO difference endpoint, planned *post hoc* contrasts included T2-T1, T3-T2, T4-T2, and T4-T3 to characterize the emergence, persistence, or recovery of time-related changes. Unless otherwise specified, *p* values reported below refer to the LMM main effect of timepoint. As sensitivity analyses, generalized estimating equation (GEE) models were additionally fitted for the delta sub-band, aperiodic-association, and connectivity analyses, with results reported in Supplementary Tables S1, S2, and S3.

## Results

### Analysis space and data structure

The analysis covered canonical band power, delta sub-bands, channel-level topographies, aperiodic spectral parameters, complexity/entropy metrics, EEG microstate parameters, and delta-band connectivity measures. The primary inference models treated timepoint as a fixed effect and subject as a random intercept. Given the exploratory nature and small sample size of this study, we emphasize effect sizes and the consistency of patterns across regions, rather than statistical significance alone. In the following sections, *p* values refer to the LMM main effect of timepoint, and FDR values reflect Benjamini–Hochberg correction within the same feature family. Reporting the full analysis space was intended to improve transparency and reduce selective emphasis on significant findings.

### Primary endpoint: the delta EC–EO difference showed the most consistent longitudinal effect

Among canonical frequency-band features, the most consistent longitudinal effect was observed for the delta EC–EO difference. Region-level LMMs showed FDR-significant main effects of timepoint in parietal, frontal, whole-brain, and occipital summaries (Table 2). The strongest overall effect was observed in the parietal region (*p* = 0.000191, FDR = 0.00573), followed by the frontal region (*p* = 0.00147, FDR = 0.0196), whole-brain summary (*p* = 0.00196, FDR = 0.0196), and occipital region (*p* = 0.00394, FDR = 0.0296). Other canonical frequency bands did not show an equally consistent FDR-corrected spatial pattern. The omnibus effect sizes (Cramér's V) for the four key regions were 0.31 (parietal), 0.28 (frontal and whole-brain), and 0.27 (occipital), corresponding to medium-to-large effects.

**Table 2:** Regional linear mixed-effects model main effects of timepoint for the delta EC–EO difference.

Region	*n*	LMM *χ^2^*	df	*p*	FDR	*V*
Parietal	63	18.73	3	0.000191	0.00573	0.31
Frontal	63	15.29	3	0.00147	0.0196	0.28
Whole-brain	63	14.85	3	0.00196	0.0196	0.28
Occipital	63	13.34	3	0.00394	0.0296	0.27
Temporal	63	10.14	3	0.0175	0.0747	0.23
Central	63	7.85	3	0.0496	0.124	0.20

Figure 1A presents the regional delta EC–EO difference heatmap across the four timepoints. Delta EC–EO difference log power was generally more negative at T3 and T4 across regions. To decompose these changes, we examined separate longitudinal trajectories for EO and EC delta power (Supplementary Table S4). EC delta log power showed a decreasing trend across T2, T3, and T4 that reached significance in GEE sensitivity models (global *p* = 5.1 × 10^−5^; occipital p = 2.0 × 10^−10^; parietal *p* = 1.1 × 10^−4^; frontal *p* = 0.023), whereas the corresponding LMM likelihood-ratio tests did not reach significance (all *p* > 0.05). EO delta log power showed no consistent time effects in either framework (all LMM *p* > 0.05; GEE p > 0.05 except parietal, *p* = 0.019). These results indicate that the progressive negative shift of the delta EC–EO difference was driven primarily by decreases in EC delta power, with a smaller and less consistent contribution from EO-side changes. Figure 2 displays the longitudinal trajectories of delta EC–EO difference in the four significant regions, showing progressive negative shifts from T2 through T4. Individual variability was evident (Supplementary Figure S1), with some participants following the group-level direction and others showing smaller changes or phase-specific fluctuations.

**Figure 1:**
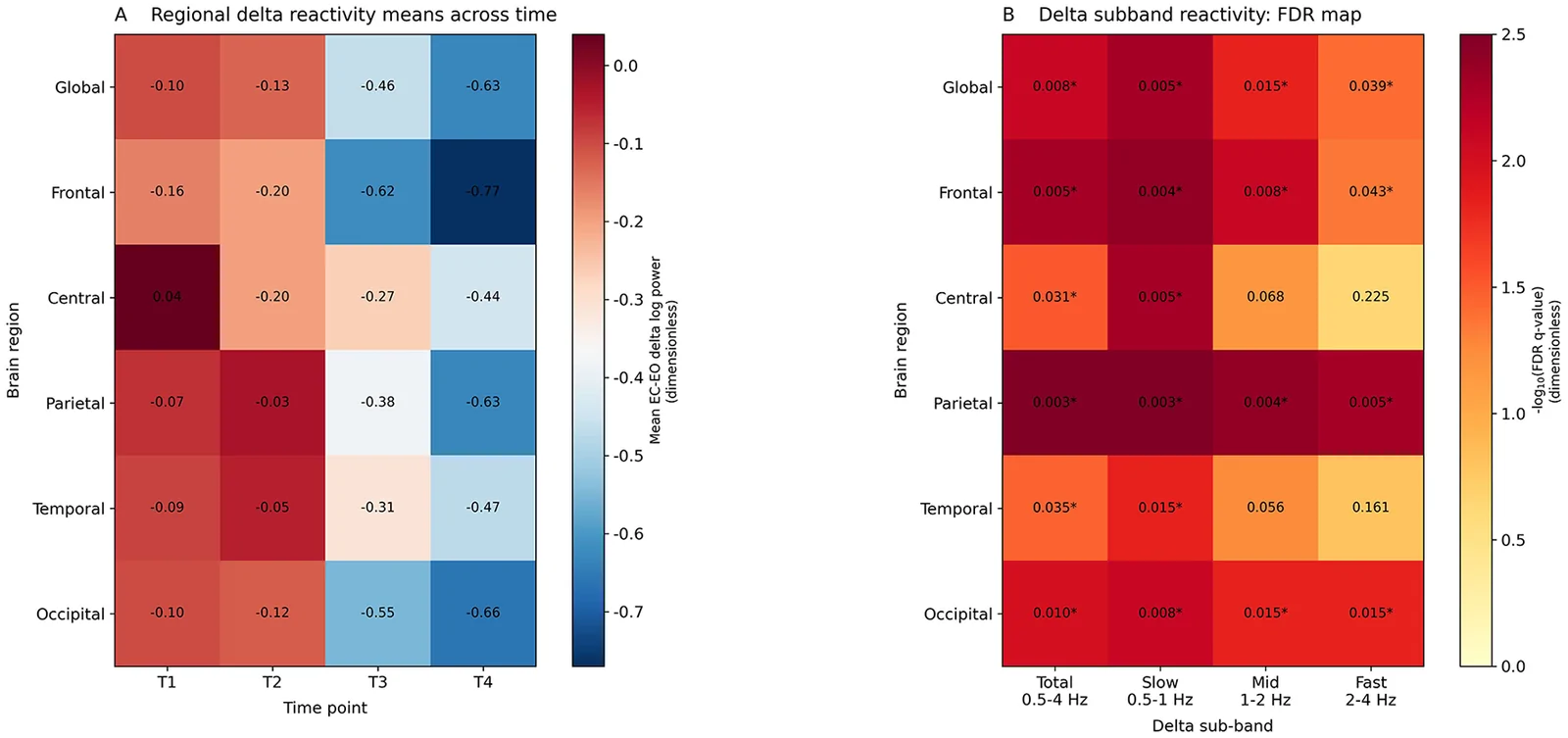
Regional delta EC–EO difference across four expedition timepoints and delta sub-band significance. **(A)** Heatmap of delta EC–EO difference (EC minus EO delta log power) across six brain regions at T1–T4. More negative values were observed at T3 and T4, particularly in parietal, frontal, whole-brain, and occipital regions. **(B)** FDR-corrected significance matrix for delta sub-band delta EC–EO difference. Each cell represents the LMM main effect of timepoint for a given region–sub-band combination. Red cells indicate FDR <0.05. The parietal region showed the most consistent significance across total delta (0.5–4 Hz), slow delta (0.5–1 Hz), mid delta (112 Hz), and fast delta (2–4 Hz), suggesting that the canonical delta EC–EO difference effect was not confined to a single narrow sub-band. EC–EO, eyes closed/open; LMM, linear mixed-effects model; FDR, false discovery rate.

**Figure 2:**
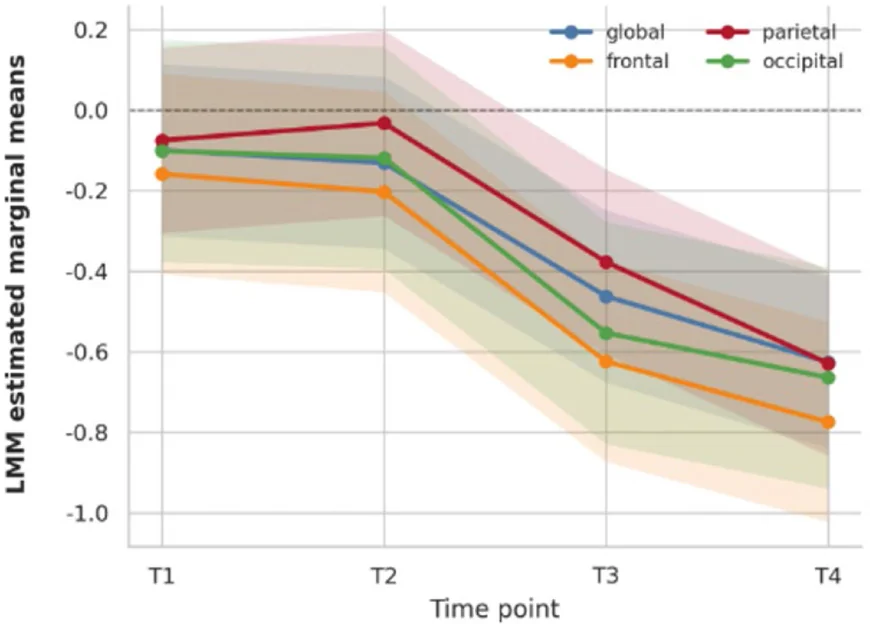
Longitudinal delta EC–EO difference trajectories in significant regions. LMM-estimated marginal means of the delta EC–EO difference across T1–T4 for parietal, frontal, whole-brain, and occipital regions, with 95% confidence intervals (shaded bands) reflecting between-subject variability in this small cohort. Planned post hoc contrasts indicated that the delta EC–EO difference did not change between T1 and T2, decreased significantly between T2 and T3 (all four regions, FDR <0.05), remained significantly different from T2 at T4, and did not change significantly between T3 and T4 (Supplementary Table S6). LMM main effects of timepoint: parietal (*p* = 0.000191, FDR = 0.00573), frontal (*p* = 0.00147, FDR = 0.0196), whole-brain (*p* = 0.00196, FDR = 0.0196), and occipital (*p* = 0.00394, FDR = 0.0296). LMM, linear mixed-effects model; FDR, false discovery rate.

Planned post hoc contrasts for the four significant regions are summarized in Supplementary Table S5. The T2–T1 contrasts were small and not significant in any region (all FDR > 0.8), indicating that the delta EC–EO difference did not change between baseline and the pre-polar-night assessment. In contrast, the T3–T2 contrasts were consistently negative and reached FDR-corrected significance in all four regions (parietal estimate = −0.345, 95% CI [−0.617, −0.073], FDR = 0.028; frontal estimate = −0.421, 95% CI [−0.773, −0.069], FDR = 0.033; occipital estimate = −0.435, 95% CI [−0.792, −0.078], FDR = 0.033; whole-brain estimate = −0.331, 95% CI [−0.633, −0.030], FDR = 0.047), indicating that the main change occurred between the pre-polar-night assessment and the early post-polar-night assessment. The T4–T2 contrasts remained significant in all four regions (e.g., parietal estimate = −0.597, 95% CI [−0.868, −0.325], FDR = 4.0 × 10^−4^, Cohen's dz = −1.93), indicating persistence of the difference at the austral-summer assessment. The T4–T3 contrasts were not FDR-significant (parietal estimate = −0.252, FDR = 0.098), indicating that the delta EC–EO difference did not increase further between the early post-polar-night and austral-summer assessments.

### Band-specific topographies and delta sub-bands

Channel-level EO/EC topographies showed that the delta band was generally more negative during T3 and T4, consistent with the regional delta EC–EO difference findings. Alpha-band EO/EC differences showed a relatively stable positive posterior distribution across timepoints. Theta, beta, and gamma topographies were more spatially dispersed and did not show a longitudinal pattern as consistent as delta EC–EO difference (Figure 3).

**Figure 3:**
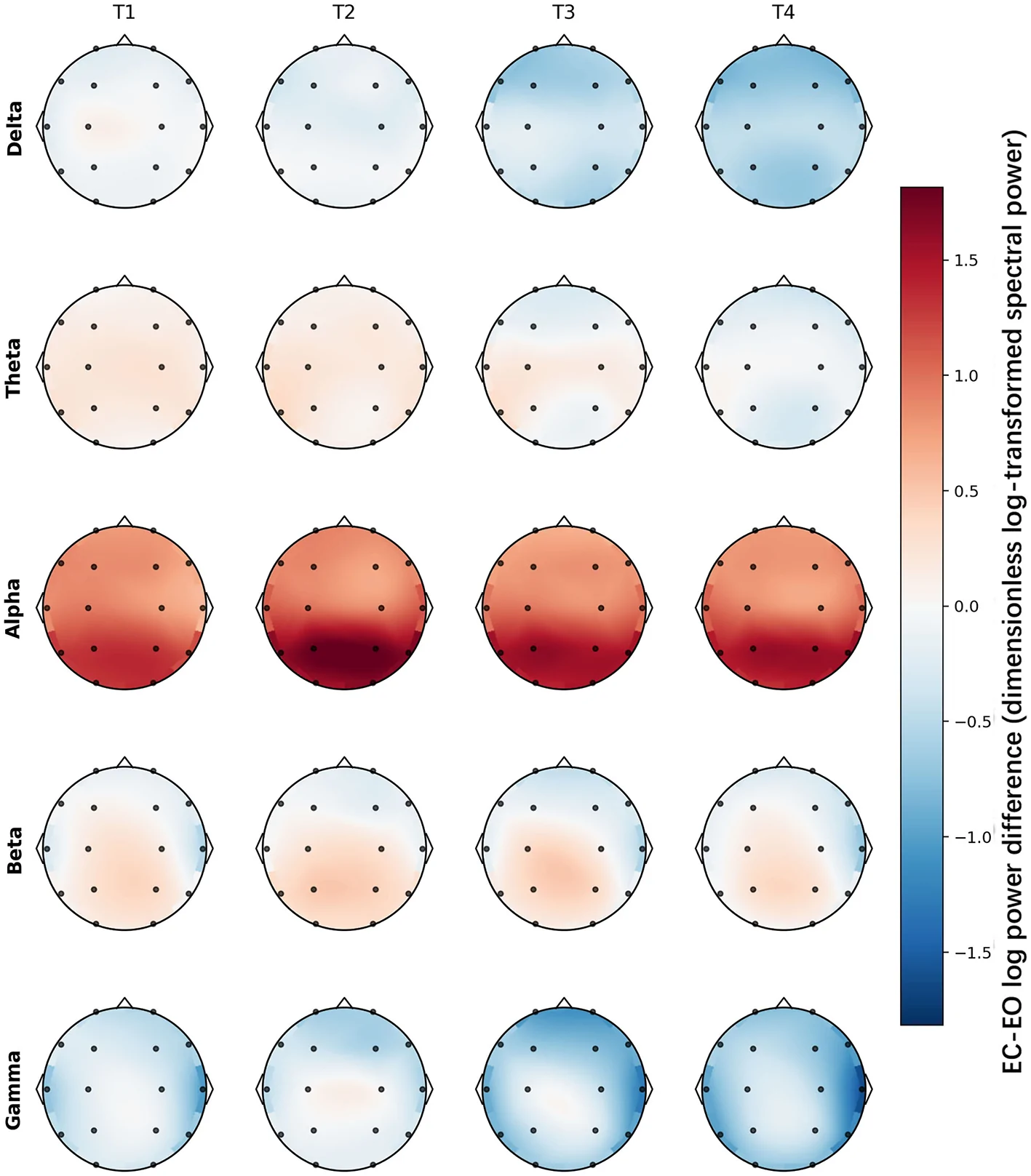
Band-specific EC–EO delta scalp topographies. Topographic maps of EC minus EO log power difference across canonical frequency bands (delta, theta, alpha, beta, gamma) at each timepoint. The delta band exhibited the most consistent negative topography during T3 and T4, with a broad spatial distribution. Alpha band showed a relatively stable positive posterior distribution across timepoints. Theta, beta, and gamma topographies were more spatially dispersed without consistent longitudinal patterns. Electrode positions follow the 10–20 system (mapped to standard_1020 montage for visualization). EC, eyes closed; EO, eyes open.

Delta sub-band analyses further indicated that the low-frequency effect extended across multiple delta ranges (Figure 1B, full results in Supplementary Table S1). Among the 23 delta sub-band reactivity tests with results available, 20 reached FDR <0.05. The parietal region was the most consistent region, with total delta, slow delta, mid delta, and fast delta all showing FDR-corrected significance. The strongest results included parietal total delta (*p* = 0.000218, FDR = 0.00261), parietal slow delta (*p* = 0.000177, FDR = 0.00261), parietal mid delta (*p* = 0.000496, FDR = 0.00397), frontal slow delta (*p* = 0.000738, FDR = 0.00443), whole-brain slow delta (*p* = 0.001245, FDR = 0.00512), and frontal total delta (*p* = 0.001364, FDR = 0.00512). These results suggest that the canonical delta EC–EO difference time effect was not driven by a single narrow delta sub-band.

### Aperiodic spectral parameters

To test whether delta EC–EO difference changes were independent of broad spectral background shifts, we examined the statistical association between delta EC–EO difference and aperiodic spectral parameters using joint linear mixed-effects models. These models included delta EC–EO difference as the dependent variable, aperiodic offset and exponent as simultaneous fixed-effect predictors, timepoint as a categorical covariate, and subject as a random intercept. The aim of this analysis was to quantify the degree to which delta EC–EO difference co-varies with the overall 1/f spectral shape, rather than to test causal relationships. This model specification differs from the primary timepoint-only model in that aperiodic offset and exponent enter as simultaneous fixed-effect predictors, with timepoint retained as a categorical covariate; the full model was delta EC–EO difference ~ aperiodic offset + aperiodic exponent + timepoint + (1 | subject).

Joint models showed stable statistical associations between delta EC–EO difference and aperiodic offset and exponent. Across regions, offset coefficients were positive and exponent coefficients were negative, and both predictors reached statistical significance in all regions. In the whole-brain model, offset beta = 1.059 (*p* = 6.75 × 10^−16^) and exponent beta = −0.594 (*p* = 8.12 × 10^−8^). In the occipital model, offset beta = 1.356 (*p* = 2.94 × 10^−51^) and exponent beta = −0.690 (*p* = 5.58 × 10^−16^). In the parietal model, offset beta = 1.018 (*p* = 1.23 × 10^−37^) and exponent beta = −0.359 (*p* = 1.68 × 10^−6^). Full coefficient summaries are provided in Supplementary Figure S2 and Table S2.

These findings indicate close statistical coupling between delta EC–EO difference and aperiodic spectral shape. Because delta power, offset, and exponent were estimated from the same power spectra, this result is reported as a spectral-shape association and is not interpreted as evidence of a causal mechanism.

### Complexity, entropy, and spectral morphology

Complexity, entropy, and spectral morphology features showed a small number of secondary time effects. In within-condition models, no complexity, entropy, or spectral morphology metric reached FDR <0.05. In EO/EC difference models, one metric reached FDR <0.05: occipital sample entropy (*p* = 0.00209, FDR = 0.0461).

The timepoint means for occipital sample entropy reactivity were T1 = −0.0768, T2 = 0.0138, T3 = 0.0372, and T4 = 0.0296 (Supplementary Figure S3). This metric was negative at T1, whereas T2-T4 means were close to or above zero, suggesting a time-related change in EO/EC complexity difference. Other complexity metrics, including permutation entropy and Lempel-Ziv complexity, did not show FDR-corrected effects as stable as sample entropy.

### EEG microstate analyses

EEG microstate analyses did not show robust FDR-corrected longitudinal effects. The within-condition microstate models included 24 tests, and the EO/EC difference microstate models included 12 tests; none reached FDR <0.05. In the within-condition models, the smallest uncorrected p value was observed for EC MS4 mean duration (*p* = 0.0353, FDR = 0.621). In the reactivity models, the smallest uncorrected p value was observed for MS1 occurrence (*p* = 0.0208, FDR = 0.250). Thus, EEG microstate parameters should be reported as negative or secondary findings in the present dataset.

### Functional connectivity

Given the low-density 16-channel montage, small sample size, and known susceptibility of scalp connectivity measures to volume conduction and common-reference effects, all connectivity results should be interpreted as strictly exploratory. Delta-band functional connectivity showed several exploratory time-related effects (Table S3). In the global connectivity analysis, EO/EC delta dwPLI reactivity showed a main effect of timepoint (p = 0.00262, FDR = 0.00524). Figure 4 displays the global delta dwPLI reactivity across timepoints. The timepoint means at T1, T2, T3, and T4 were 0.00199, 0.00392, −0.02073, and 0.01171, respectively, suggesting that global delta phase-synchrony reactivity varied across the four timepoints, with the T3 mean differing in direction from the other timepoints.

**Figure 4:**
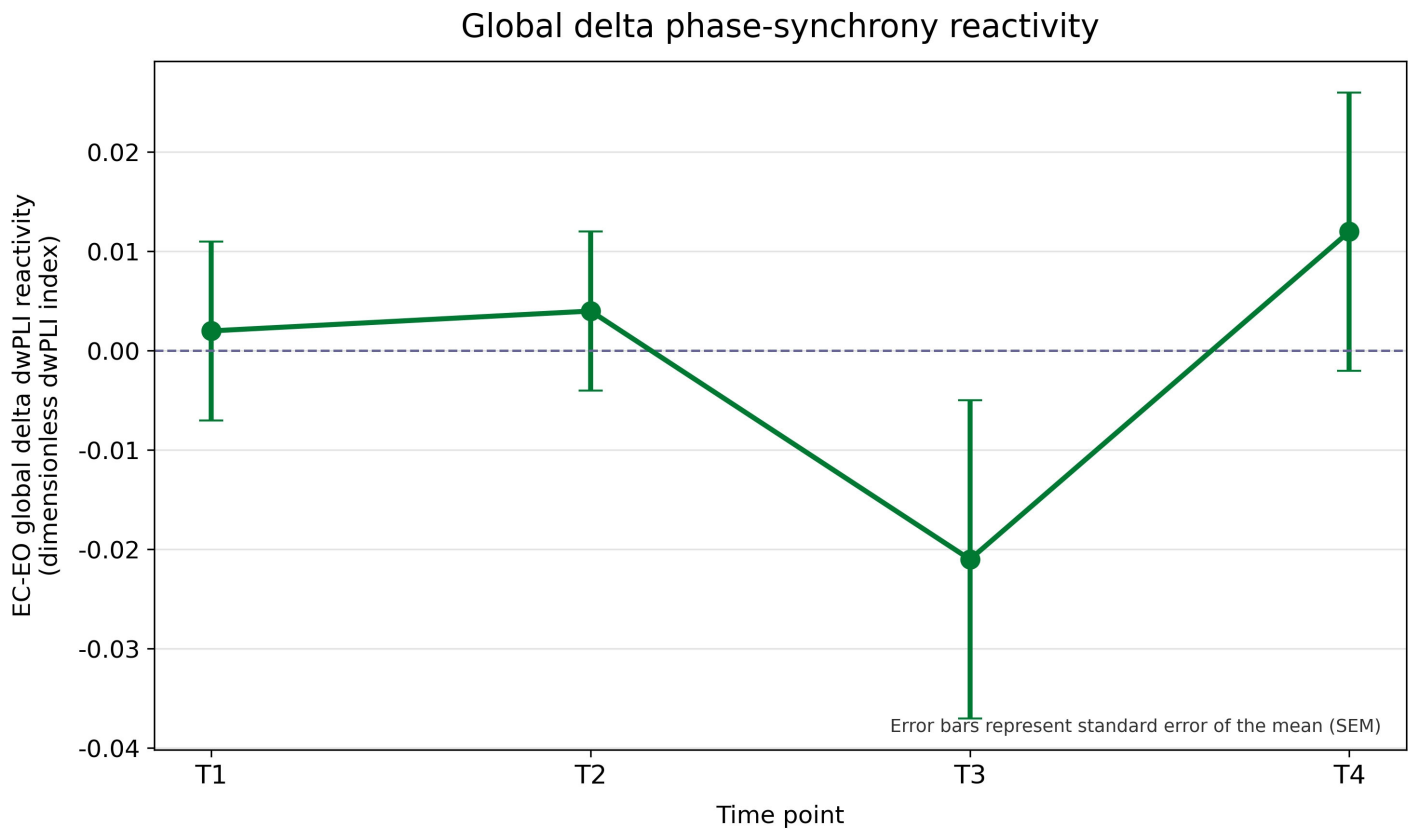
Global delta dwPLI reactivity across timepoints. EO/EC delta dwPLI reactivity (EC minus EO dwPLI) showed a significant main effect of timepoint (*p* = 0.00262, FDR = 0.00524). Timepoint means were T1 = 0.00199, T2 = 0.00392, T3 = −0.02073, and T4 = 0.01171, indicating that global delta phase-synchrony reactivity varied across the expedition timeline, with the T3 mean differing in direction from other timepoints. Error bars represent standard errors of the mean. dwPLI, debiased weighted phase-lag index; FDR, false discovery rate.

In region-pair connectivity analyses, FDR-corrected effects were concentrated in EC PLV. Significant region pairs included parietal-parietal (*p* = 0.000204, FDR = 0.00307), parietal-temporal (*p* = 0.00108, FDR = 0.00811), parietal-occipital (*p* = 0.00237, FDR = 0.00887), temporal-temporal (*p* = 0.00200, FDR = 0.00887), occipital-occipital (*p* = 0.00370, FDR = 0.0111), and temporal-occipital (*p* = 0.0127, FDR = 0.0317). The full region-pair significance matrix is provided in Supplementary Figure S4. Given the low-density EEG montage, small sample size, and susceptibility of connectivity estimates to volume conduction and reference effects, these connectivity findings should be interpreted as exploratory.

## Discussion

This exploratory longitudinal resting-state EEG study found that delta EC–EO difference was the most stable EEG signal changing across the Antarctic expedition timeline. Four findings emerged. First, delta EC–EO difference showed the most consistent LMM main effects of timepoint in parietal, frontal, whole-brain, and occipital regions. Second, delta sub-band analyses suggested that the low-frequency EO/EC differentiation effect extended across slow, mid, and fast delta ranges. Third, aperiodic spectral parameters, complexity/entropy metrics, and functional connectivity provided secondary exploratory signals. Fourth, EEG microstate parameters did not show robust FDR-corrected longitudinal effects.

The delta EC–EO difference findings should be interpreted as changes in resting-state condition differentiation or state reactivity. Because delta EC–EO difference was defined as EC delta log power minus EO delta log power, the endpoint represents a difference between two resting-state conditions rather than absolute delta activity in EC recordings. The more evident T3/T4 pattern relative to T2 or earlier stages is consistent with altered EO/EC low-frequency differentiation during and after the polar-night period. However, this result does not establish that the polar night itself produced the observed EEG pattern, and it does not exclude contributions from sleep, fatigue, mood, light exposure, workload, seasonal timing, or other expedition-related factors.

From a physiological perspective, the longitudinal delta EC–EO difference pattern may reflect arousal-dependent changes in low-frequency modulation and resting-state reactivity. Circadian disruption, altered light exposure, isolation, and the broader polar-night context might contribute to this pattern (Castillo et al., 2023; Paul et al., 2015). These interpretations remain tentative because the current dataset does not include synchronized behavioral, sleep, endocrine, light-exposure, or workload covariates. The findings are therefore best viewed as consistent with changes in state regulation rather than as direct evidence for a specific mechanism.

Importantly, as acknowledged in the limitations, this study cannot determine whether the observed EEG changes are specifically caused by polar night, or whether they reflect cumulative effects of time spent in isolation, workload, sleep disruption, seasonal timing, or other expedition-related factors. Without synchronized measures of light exposure, sleep quality, fatigue, and circadian phase, the specific mechanisms underlying the delta EC–EO difference trajectory remain speculative.

Taken together, the present findings provide evidence of longitudinal changes in EEG state reactivity across expedition stages; they do not, by themselves, establish neurophysiological adaptation to the polar night, which would require synchronized behavioral, physiological, circadian, and environmental measures. We therefore frame the primary contribution of this study as exploratory evidence that resting-state EEG reactivity changes longitudinally during an Antarctic expedition, and as a basis for future studies with larger samples and multi-domain measurements.

Furthermore, the delta EC–EO trajectories exhibited notable inter-individual variability. Accordingly, the observed group-level pattern represents a time-related trend across this small longitudinal cohort, rather than a uniform directional change shared by all participants.

The aperiodic spectral findings suggest that delta EC–EO difference was statistically coupled with broad spectral shape. This result is consistent with the idea that conventional frequency-band power can be influenced by aperiodic background activity. However, because delta power, aperiodic offset, and aperiodic exponent were all estimated from the same power spectra, the observed associations should not be interpreted as causal evidence. Similarly, the complexity/entropy and connectivity results provide candidate features for future work but should not be treated as primary conclusions.

The negative EEG microstate results are also informative. In the present low-density EEG dataset, mean duration, occurrence, and coverage did not provide robust FDR-corrected evidence for longitudinal time effects. This does not rule out the possibility that high-density EEG, longer recordings, or alternative microstate modeling strategies could identify dynamic topographic changes in future studies. In the current manuscript, however, microstate findings should remain secondary and negative.

The primary methodological contribution of this study is the provision of a reproducible longitudinal EEG analysis framework for extreme-environment research. The framework integrates spectral power, EO/EC reactivity, aperiodic spectral parameters, complexity/entropy metrics, EEG microstates, and delta-band connectivity while using LMMs to account for repeated measures within participants. For future Antarctic, spaceflight-analog, submarine, or other isolated extreme-environment studies, such a framework may support quantitative neurophysiological monitoring, provided that larger samples and richer behavioral and physiological metadata become available. The use of EO/EC difference metrics, in particular, may help reduce interindividual baseline variability in small-sample longitudinal studies, although the statistical advantage of this approach relative to absolute power models remains to be formally quantified.

### Potential translational implications and future directions

The longitudinal delta EC–EO difference pattern identified here may have several translational implications, but all remain hypothetical and require formal validation in larger studies with independent physiological and behavioral measures.

First, if its sensitivity, specificity, and test-retest reliability are confirmed, delta EC–EO difference could serve as a complementary candidate index in neurohealth surveillance protocols for workers in chronically isolated and light-deprived settings, such as Antarctic expeditioners, long-duration spaceflight crews, and submarine personnel. Currently, occupational monitoring in these groups relies heavily on subjective scales and cognitive tests, which are vulnerable to reporting bias and practice effects. A candidate EEG index of resting-state differentiation could potentially aid early detection of subclinical alterations in state regulation, but its utility as an early-detection marker remains to be validated.

Second, delta EC–EO difference may provide an additional dimension for phenotypic stratification in sleep and circadian rhythm disorders, such as seasonal affective disorder, delayed sleep-wake phase disorder, and chronic insomnia. These conditions are often associated with altered arousal regulation and impaired resting-state differentiation, but conventional clinical EEG focuses primarily on alpha reactivity. The delta EC–EO difference metric could potentially help distinguish between fatigue-predominant and anxiety-predominant insomnia phenotypes; however, this hypothesis requires direct testing in clinical cohorts.

Third, the observed association between delta EC–EO difference and time spent in an extreme light-deprived environment is consistent with theories implicating arousal system dysfunction in chronic fatigue. If validated, this metric could contribute to the multidimensional assessment of fatigue-related conditions, but it cannot currently be used to objectively grade fatigue severity or monitor treatment response.

Fourth, delta EC–EO difference could potentially be explored as a quantitative endpoint for non-pharmacological interventions targeting circadian disruption and arousal dysregulation, such as bright light therapy and cognitive behavioral therapy for insomnia. However, its responsiveness to intervention and its correlation with clinical outcomes have not been evaluated.

Furthermore, the analytical framework established in this study provides a methodological reference for longitudinal clinical EEG research with limited sample sizes. The hierarchical analytical strategy featuring a pre-specified primary endpoint, multiple secondary feature families, and full disclosure of negative findings, combined with the statistical approach of linear mixed-effects models with false discovery rate correction applied within each feature family, constitutes a replicable paradigm for exploratory clinical EEG studies. Small sample sizes are common in clinical research involving rare diseases or special occupational cohorts. The present study illustrates a framework in which eyes-closed/eyes-open difference metrics may reduce interindividual baseline variability relative to absolute power under a single condition; however, a formal statistical comparison with models based on absolute power would be required to confirm any improvement in statistical power. Full reporting of the entire analytical space and all null findings also mitigates selective reporting bias and enhances the reproducibility and credibility of clinical EEG studies, offering methodological insights for similar exploratory clinical investigations.

### Limitations

This study has several limitations. The small sample size (*n* = 16), typical of Antarctic field research, limits statistical power and effect-size precision. The 16-channel low-density montage restricts spatial resolution and source localization. The lack of synchronized physiological, behavioral, and environmental covariates prevents disentangling polar-night-specific effects from general longitudinal changes, and drowsiness or fatigue confounding cannot be ruled out. Connectivity analyses are limited by partial sample loss and inherent vulnerabilities of scalp EEG to volume conduction and common-reference effects. Several additional methodological caveats should be acknowledged. First, the primary within-participant analysis retained all 63 readable participant-timepoint pairs, including 11 pairs in which one condition had been flagged for a low epoch count (3–18 usable epochs); although this preserved the paired longitudinal structure, spectral estimates from the shortest recordings are less stable than those from longer recordings and may have increased variability for those observations. Second, several acquisition parameters that could not be recovered from the original project files (e.g., amplifier model, electrode impedance thresholds, and online filter settings) are not described in the Methods; this limits the completeness of the acquisition protocol for exact replication, although the preprocessing and analysis pipeline itself is described in full. Third, the claim that EC–EO difference metrics improve statistical power relative to absolute-power models requires a formal model comparison that was not performed here. Finally, this exploratory observational design cannot support causal or mechanistic conclusions.

## Conclusion

In this exploratory longitudinal study of 16 Antarctic expeditioners, resting-state EEG was recorded across four phases: pre-departure, pre-polar-night, early post-polar-night, and austral summer. Delta-band eyes-closed/open differentiation (delta EC–EO difference) was the most robust finding, showing progressively negative shifts across the expedition stages in parietal, frontal, whole-brain, and occipital regions, with the most pronounced changes observed between the pre-polar-night and subsequent assessments. Mild, less consistent longitudinal changes were seen in aperiodic spectral metrics, occipital sample entropy, and delta-band functional connectivity, while EEG microstates showed no stable effects. These results provide exploratory evidence of longitudinal alterations in resting-state EEG reactivity across the stages of an Antarctic expedition. Whether these changes specifically reflect the polar night or other expedition-related factors, and whether they have utility as monitoring indices, remain to be confirmed in larger studies with higher-density recordings and synchronized behavioral, physiological, and environmental measures.

## Significance

This study provides a candidate, exploratory neurophysiological framework for monitoring state-regulation changes in extreme, isolated environments, with potential applications in occupational and sleep medicine pending further validation.

## Data Availability

The raw data supporting the conclusions of this article will be made available by the authors, without undue reservation.
