# Supplementary material for: Longitudinal changes in resting-state EEG eyes-closed/open delta differentiation during an Antarctic expedition
Source: Front Neurosci. 2026 Oct 2;20:1944325. doi: 10.3389/fnins.2026.1944325 (PMC13672630; doi:10.3389/fnins.2026.1944325)
Supplement: Supplementary file 1 [file Supplementary_file_1.docx]

**Supplementary materials**

**Table S1. Complete LMM and GEE results for delta sub-band delta EC–EO difference across all region–sub-band combinations.**

| **Region** | **Sub-band** | **n** | **LMM p** | **LMM FDR** | **GEE p** | **GEE FDR** |
| --- | --- | --- | --- | --- | --- | --- |
| **Parietal** | Total | 63 | 0.000218 | 0.00261 | 3.81×10⁻¹⁵ | 4.57×10⁻¹⁴ |
|  | Slow | 63 | 0.000177 | 0.00261 | 2.54×10⁻¹³ | 2.03×10⁻¹² |
|  | Mid | 63 | 0.000496 | 0.00397 | 1.62×10⁻¹⁷ | 3.88×10⁻¹⁶ |
|  | Fast | 63 | 0.00176 | 0.00529 | 1.85×10⁻⁴ | 5.48×10⁻⁴ |
| **Frontal** | Total | 63 | 0.00136 | 0.00512 | 2.05×10⁻⁴ | 5.48×10⁻⁴ |
|  | Slow | 63 | 0.000738 | 0.00443 | 8.09×10⁻⁷ | 4.85×10⁻⁶ |
|  | Mid | 63 | 0.00351 | 0.00836 | 5.21×10⁻⁴ | 8.81×10⁻⁴ |
|  | Fast | 63 | 0.0356 | 0.0427 | 0.0981 | 0.0981 |
| **Central** | Slow | 63 | 0.00149 | 0.00512 | 5.50×10⁻⁴ | 8.81×10⁻⁴ |
|  | Total | 63 | 0.0223 | 0.0314 | 6.97×10⁻³ | 8.36×10⁻³ |
|  | Mid | 63 | 0.0627 | 0.0684 | 0.0190 | 0.0217 |
| **Occipital** | Slow | 63 | 0.00383 | 0.00836 | 1.92×10⁻⁴ | 5.48×10⁻⁴ |
|  | Total | 63 | 0.00500 | 0.0100 | 2.75×10⁻⁴ | 5.86×10⁻⁴ |
|  | Mid | 63 | 0.00874 | 0.0152 | 1.65×10⁻³ | 2.33×10⁻³ |
|  | Fast | 63 | 0.00956 | 0.0152 | 5.75×10⁻³ | 7.60×10⁻³ |
| **Temporal** | Slow | 63 | 0.0102 | 0.0152 | 2.30×10⁻⁴ | 5.51×10⁻⁴ |
|  | Total | 63 | 0.0265 | 0.0354 | 7.80×10⁻⁵ | 3.12×10⁻⁴ |
|  | Mid | 63 | 0.0493 | 0.0563 | 2.93×10⁻⁴ | 5.86×10⁻⁴ |
|  | Fast | 63 | 0.154 | 0.161 | 6.02×10⁻³ | 7.60×10⁻³ |
| **Whole-brain** | Slow | 63 | 0.00124 | 0.00512 | 1.50×10⁻⁶ | 7.21×10⁻⁶ |
|  | Total | 63 | 0.00312 | 0.00833 | 3.43×10⁻⁴ | 6.33×10⁻⁴ |
|  | Mid | 63 | 0.00909 | 0.0152 | 1.39×10⁻³ | 2.08×10⁻³ |
|  | Fast | 63 | 0.0311 | 0.0393 | 0.0628 | 0.0657 |

*Note:* Delta EC–EO difference was defined as eyes-closed minus eyes-open delta log power. Sub-band abbreviations: Total = 0.5–4 Hz; Slow = 0.5–1 Hz; Mid = 1–2 Hz; Fast = 2–4 Hz. LMM, linear mixed-effects model; GEE, generalized estimating equation; FDR, false discovery rate (Benjamini–Hochberg correction within the delta sub-band reactivity feature family). Significant FDR-corrected effects (FDR < 0.05) are shown in bold. Empty cells in the Region column indicate the same region as the row above.

**Table S2. Joint models of delta EC–EO difference with aperiodic offset and exponent: full regional coefficient summaries.**

| Region | n | LMM Offset β | LMM Offset p | LMM Exponent β | LMM Exponent p | GEE Offset β | GEE Offset p | GEE Exponent β | GEE Exponent p |
| --- | --- | --- | --- | --- | --- | --- | --- | --- | --- |
| Occipital | 63 | 1.356 | 2.94×10⁻⁵¹ | –0.690 | 5.58×10⁻¹⁶ | 1.355 | 6.29×10⁻⁴³ | –0.689 | 2.27×10⁻¹² |
| Parietal | 63 | 1.018 | 1.23×10⁻³⁷ | –0.359 | 1.68×10⁻⁶ | 1.018 | 5.35×10⁻³⁸ | –0.359 | 8.91×10⁻⁸ |
| Whole-brain | 63 | 1.059 | 6.75×10⁻¹⁶ | –0.594 | 8.12×10⁻⁸ | 1.027 | 1.31×10⁻¹² | –0.588 | 7.25×10⁻⁷ |
| Temporal | 63 | 0.984 | 6.69×10⁻¹⁷ | –0.478 | 9.47×10⁻⁶ | 0.982 | 2.74×10⁻¹⁴ | –0.478 | 2.02×10⁻⁵ |
| Frontal | 63 | 0.958 | 4.76×10⁻⁷ | –0.588 | 8.63×10⁻⁵ | 0.897 | 6.65×10⁻⁸ | –0.574 | 4.12×10⁻⁵ |
| Central | 63 | 0.724 | 7.73×10⁻⁷ | –0.480 | 1.36×10⁻³ | 0.730 | 1.22×10⁻⁴ | –0.507 | 7.17×10⁻⁵ |

*Note:* Joint models included delta EC–EO difference as the dependent variable and aperiodic offset + exponent as simultaneous predictors, with timepoint as a categorical fixed effect and subject as a random intercept. LMM, linear mixed-effects model; GEE, generalized estimating equation. All p values shown are < 0.05 and are therefore formatted in bold for readability; the LMM predictor block p values (not shown) were also significant in all regions (all p < 0.001). Offset coefficients are positive and exponent coefficients are negative across all regions, indicating consistent spectral-shape coupling with delta EC–EO difference.

**Table S3.** Full delta-band functional connectivity results.

| Analysis | Method | Region Pair | n | LMM p | LMM FDR | GEE p | GEE FDR |
| --- | --- | --- | --- | --- | --- | --- | --- |
| EC–EO global | dwPLI | Global | 63 | 0.00262 | 0.00524 | 0.0252 | 0.0504 |
| EC region-pair | PLV | Parietal–Parietal | 61 | 0.000204 | 0.00307 | 1.35×10⁻⁵ | 6.76×10⁻⁵ |
| EC region-pair | PLV | Parietal–Temporal | 61 | 0.00108 | 0.00811 | 2.80×10⁻⁶ | 2.10×10⁻⁵ |
| EC region-pair | PLV | Parietal–Occipital | 61 | 0.00237 | 0.00887 | 2.60×10⁻⁵ | 9.77×10⁻⁵ |
| EC region-pair | PLV | Temporal–Temporal | 61 | 0.00200 | 0.00887 | 0.00830 | 0.0208 |
| EC region-pair | PLV | Occipital–Occipital | 61 | 0.00370 | 0.0111 | 1.30×10⁻⁶ | 1.95×10⁻⁵ |
| EC region-pair | PLV | Temporal–Occipital | 61 | 0.0127 | 0.0317 | 3.94×10⁻³ | 0.0118 |
| EC region-pair | PLV | Central–Occipital | 61 | 0.0236 | 0.0506 | 0.0132 | 0.0284 |
| EC region-pair | dwPLI | Temporal–Occipital | 63 | 0.0198 | 0.0991 | 4.78×10⁻⁴ | 7.17×10⁻³ |

*Note:* PLV, phase-locking value; dwPLI, debiased weighted phase-lag index; LMM, linear mixed-effects model; GEE, generalized estimating equation; FDR, false discovery rate. Significant LMM FDR-corrected effects (LMM FDR < 0.05) are shown in bold. LMM χ² values are omitted for brevity (all models had df = 3 for the timepoint fixed effect).

**Table S4.** Condition-level LMM and GEE main effects of timepoint for EC and EO delta log power across all regions.

| Condition | Region | n | LMM p | GEE p | GEE FDR |
| --- | --- | --- | --- | --- | --- |
| EC | Whole-brain | 64 | 0.243 | 5.1 × 10⁻⁵ | 6.5 × 10⁻⁴ |
| EC | Frontal | 64 | 0.219 | 0.0231 | 0.0694 |
| EC | Central | 64 | 0.431 | 0.213 | 0.304 |
| EC | Parietal | 64 | 0.112 | 1.1 × 10⁻⁴ | 8.2 × 10⁻⁴ |
| EC | Temporal | 64 | 0.285 | 6.5 × 10⁻⁵ | 6.5 × 10⁻⁴ |
| EC | Occipital | 64 | 0.261 | 2.0 × 10⁻¹⁰ | 6.0 × 10⁻⁹ |
| EO | Whole-brain | 63 | 0.139 | 0.533 | 0.714 |
| EO | Frontal | 63 | 0.109 | 0.484 | 0.714 |
| EO | Central | 63 | 0.232 | 0.556 | 0.714 |
| EO | Parietal | 63 | 0.0632 | 0.0192 | 0.289 |
| EO | Temporal | 63 | 0.223 | 0.288 | 0.714 |
| EO | Occipital | 63 | 0.158 | 0.115 | 0.492 |

*Note:* Separate linear mixed-effects models (LMM) and generalized estimating equations (GEE) were fitted for EC and EO delta log power, with timepoint as a categorical fixed effect and subject as a random intercept. p values refer to the main effect of timepoint (LMM likelihood-ratio test; GEE Wald test). n indicates the number of readable condition recordings per the final quality-control manifest (EC 64, EO 63; one EO file was unreadable); statistics follow the original analysis output (spectral_timepoint_stats_lmm_gee.csv). The EC-side GEE decreases reached FDR-corrected significance in whole-brain, parietal, temporal, and occipital regions, whereas the corresponding LMM tests and all EO-side models did not (except EO parietal, GEE p = 0.019). These condition-level results indicate that the progressive negative shift of the delta EC–EO difference was driven primarily by EC-side decreases in delta power. LMM, linear mixed-effects model; GEE, generalized estimating equation; FDR, false discovery rate (Benjamini–Hochberg correction within each condition-level family).

**Table S5.** Planned post hoc contrasts of the delta EC–EO difference in the four key regions (LMM).

| Region | Contrast | n | Estimate | 95% CI lower | 95% CI upper | FDR | Cohen’s dz |
| --- | --- | --- | --- | --- | --- | --- | --- |
| Parietal | T2–T1 | 16 | 0.043 | −0.229 | 0.314 | 0.867 | 0.101 |
| Whole-brain | T2–T1 | 16 | −0.032 | −0.333 | 0.270 | 0.873 | −0.072 |
| Frontal | T2–T1 | 16 | −0.045 | −0.396 | 0.307 | 0.873 | −0.085 |
| Occipital | T2–T1 | 16 | −0.017 | −0.374 | 0.340 | 0.925 | −0.026 |
| Parietal | T3–T2 | 16 | −0.345 | −0.617 | −0.073 | 0.028 | −0.513 |
| Frontal | T3–T2 | 16 | −0.421 | −0.773 | −0.069 | 0.033 | −0.507 |
| Occipital | T3–T2 | 16 | −0.435 | −0.792 | −0.078 | 0.033 | −0.515 |
| Whole-brain | T3–T2 | 16 | −0.331 | −0.633 | −0.030 | 0.047 | −0.475 |
| Parietal | T4–T2 | 16 | −0.597 | −0.868 | −0.325 | 0.0004 | −1.928 |
| Whole-brain | T4–T2 | 16 | −0.495 | −0.797 | −0.193 | 0.006 | −1.234 |
| Frontal | T4–T2 | 16 | −0.572 | −0.923 | −0.220 | 0.006 | −0.889 |
| Occipital | T4–T2 | 16 | −0.546 | −0.903 | −0.189 | 0.008 | −1.130 |
| Parietal | T4–T3 | 16 | −0.252 | −0.523 | 0.020 | 0.098 | −0.386 |
| Whole-brain | T4–T3 | 16 | −0.164 | −0.465 | 0.138 | 0.383 | −0.203 |
| Frontal | T4–T3 | 16 | −0.151 | −0.502 | 0.201 | 0.507 | −0.140 |
| Occipital | T4–T3 | 16 | −0.111 | −0.468 | 0.246 | 0.651 | −0.130 |

*Note:* Planned post hoc contrasts of the delta EC–EO difference from linear mixed-effects models (LMM) with timepoint as a categorical fixed effect and subject as a random intercept. Estimates are LMM estimated differences (log-power units) between the two timepoints; 95% confidence intervals are shown; FDR refers to Benjamini–Hochberg correction applied across the four contrasts within each region; Cohen’s dz is the paired standardized effect size. T2–T1 tests change from baseline to the pre-polar-night assessment; T3–T2 tests change between the pre-polar-night and early post-polar-night assessments; T4–T2 tests persistence at the austral-summer assessment relative to the pre-polar-night assessment; T4–T3 tests further change between the early post-polar-night and austral-summer assessments. LMM, linear mixed-effects model; FDR, false discovery rate.

**Figure S1. Individual Parietal Delta EC–EO difference Longitudinal Trajectories**

*Note:* Individual participant trajectories of parietal delta EC–EO difference across T1–T4 (n = 16). The group mean trajectory (thick line) shows progressive negative shifts from T2 to T4. Interindividual variability was evident: while most participants showed trajectories consistent with the group-level direction, some exhibited smaller changes or phase-specific fluctuations (e.g., increases at T3 or T4). This variability supports reporting delta EC–EO difference as a time-related pattern at the group level rather than a uniform response across all participants. EC–EO, eyes closed/open.

**Figure S2.** LMM Regression Coefficients for Aperiodic Spectral Features Across All Regions

*Note:* Joint models showed stable statistical associations between delta EC–EO difference and aperiodic offset (positive coefficients, top panel) and exponent (negative coefficients, bottom panel) across all six regions. Error bars represent 95% confidence intervals. All offset coefficients and exponent coefficients reached statistical significance (p < 0.005 in all regions). These associations reflect spectral-shape coupling between delta-band reactivity and broadband aperiodic background activity, rather than causal evidence. LMM, linear mixed-effects model.

**Figure S3.** Occipital Sample Entropy EC–EO Differences Over Time

*Note:* Occipital sample entropy reactivity (EC minus EO) showed a significant timepoint effect (p = 0.00209, FDR = 0.0461). Means (with standard errors) at T1, T2, T3, and T4 were –0.0768, 0.0138, 0.0372, and 0.0296, respectively, suggesting a shift from negative differentiation at baseline to near-zero or positive differentiation during and after the polar-night period. Other complexity metrics did not show stable FDR-corrected effects. EC, eyes closed; EO, eyes open; FDR, false discovery rate.

**Figure S4.** EC Delta PLV Connectivity Significance Matrix for Region-Pair Analyses

*Note:* Region-pair connectivity analysis revealed FDR-corrected effects concentrated in EC PLV. Significant region pairs included parietal–parietal (p = 0.000204, FDR = 0.00307), parietal–temporal (p = 0.00108, FDR = 0.00811), parietal–occipital (p = 0.00237, FDR = 0.00887), temporal–temporal (p = 0.00200, FDR = 0.00887), occipital–occipital (p = 0.00370, FDR = 0.01110), and temporal–occipital (p = 0.01270, FDR = 0.03175). Given the low-density 16-channel montage, small sample size, and susceptibility of PLV to volume conduction and common-reference effects, these findings are exploratory. PLV, phase-locking value; EC, eyes closed; FDR, false discovery rate.
